# Vitamin D receptor expression in human bone tissue and dose-dependent activation in resorbing osteoclasts

**DOI:** 10.1038/boneres.2016.30

**Published:** 2016-10-11

**Authors:** Allahdad Zarei, Alireza Morovat, Kassim Javaid, Cameron P Brown

**Affiliations:** 1 Botnar Research Centre, Nuffield Department of Orthopaedics, Rheumatology and Musculoskeletal Science, University of Oxford, Oxford, UK; 2 Clinical Biochemistry, Oxford University Hospitals NHS Trust, Oxford, UK

## Abstract

The effects of vitamin D on osteoblast mineralization are well documented. Reports of the effects of vitamin D on osteoclasts, however, are conflicting, showing both inhibition and stimulation. Finding that resorbing osteoclasts in human bone express vitamin D receptor (VDR), we examined their response to different concentrations of 25-hydroxy vitamin D_3_ [25(OH)D_3_] (100 or 500 nmol**·**L^−1^) and 1,25-dihydroxy vitamin D_3_ [1,25(OH)_2_D_3_] (0.1 or 0.5 nmol**·**L^−1^) metabolites in cell cultures. Specifically, CD14+ monocytes were cultured in charcoal-stripped serum in the presence of receptor activator of nuclear factor kappa-B ligand (RANKL) and macrophage colony-stimulating factor (M-CSF). Tartrate-resistant acid phosphatase (TRAP) histochemical staining assays and dentine resorption analysis were used to identify the size and number of osteoclast cells, number of nuclei per cell and resorption activity. The expression of VDR was detected in human bone tissue (*ex vivo*) by immunohistochemistry and *in vitro* cell cultures by western blotting. Quantitative reverse transcription-PCR (qRT-PCR) was used to determine the level of expression of vitamin D-related genes in response to vitamin D metabolites. VDR-related genes during osteoclastogenesis, shown by qRT-PCR, was stimulated in response to 500 nmol**·**L^−1^ of 25(OH)D_3_ and 0.1–0.5 nmol**·**L^−1^ of 1,25(OH)_2_D_3_, upregulating cytochrome P450 family 27 subfamily B member 1 (*CYP27B1*) and cytochrome P450 family 24 subfamily A member 1 (*CYP24A1*). Osteoclast fusion transcripts transmembrane 7 subfamily member 4 (*tm7sf4*) and nuclear factor of activated T-cell cytoplasmic 1 (*nfatc1*) where downregulated in response to vitamin D metabolites. Osteoclast number and resorption activity were also increased. Both 25(OH)D_3_ and 1,25(OH)_2_D_3_ reduced osteoclast size and number when co-treated with RANKL and M-CSF. The evidence for VDR expression in resorbing osteoclasts *in vivo* and low-dose effects of 1,25(OH)_2_D_3_ on osteoclasts *in vitro* may therefore provide insight into the effects of clinical vitamin D treatments, further providing a counterpoint to the high-dose effects reported from *in vitro* experiments.

## Introduction

Vitamin D was discovered based on its role as an anti-rachitic agent and it is now well understood that vitamin D deficiency results in rickets in adolescents and osteomalacia in adults.^1^ Regulation of calcium and phosphate by vitamin D is widely studied and it is known to have a major role in maintaining a healthy skeleton. Conversely, vitamin D deficiency on bone *in vivo* can be compensated by the administration of calcium and phosphate. There is also growing evidence that vitamin D has a direct effect on bone cells; osteoblasts, osteocytes, and osteoclasts.^2^^–4^

*In vitro* studies have revealed that osteoblasts and osteocytes can locally synthesize the active metabolite 1,25(OH)_2_D_3_ as they express *CYP27B1*, as well as having the ability to locally catabolize vitamin D by expressing the *CYP24A1* enzyme.^5^ Peripheral blood mononuclear cell (PBMC)-derived osteoclasts have been shown to express *CYP27B1* and are capable of metabolizing 25(OH)D_3_ into 1,25(OH)_2_D_3_, suggesting that metabolism of vitamin D has a role in osteoclast differentiation.^6,7^ The level of *CYP27B1* transcript expression has been shown to increase during RANKL-induced osteoclast differentiation in PBMCs as well as in RAW 264.7 murine cell line.^7,8^

Our current understanding of the effect of 1,25(OH)_2_D_3_ on osteoclast behavior is that 1,25(OH)_2_D_3_ promotes formation, but inhibits the resorptive activity of mature osteoclasts derived from human PBMCs and murine cell line RAW 264.7.^9^ The action of vitamin D on the reduction of osteoclastogenesis is not well defined, but it has been suggested that the metabolism of 25(OH)D_3_ into 1,25(OH)_2_D_3_ reduces osteoclast resorptive capacity by the modification of cellular adhesion and a reduction in the migration of resorptive osteoclasts.^10^ It has further been proposed that the action of 1,25(OH)_2_D_3_ on bone resorption might be by an increase in the proliferation of the precursors of osteoclasts.^11^

It has been reported that VDR is not expressed by mature osteoclasts *in vivo* human tissues, but rather in osteoclast precursors.^12,13^ Stimulation of osteoclast formation by 1,25(OH)_2_D_3_ has been shown to be by the upregulation of RANKL in osteoblasts and osteocytes and a direct cell-to-cell contact between osteoclast precursors and osteoblasts.^14^ Osteoclast function can be blocked by osteoprotegerin, a decoy receptor for RANKL, and osteoprotegerin has been shown to be downregulated in osteoblasts by 1,25(OH)_2_D_3_.^15–17^

Vitamin D metabolites have been used as therapeutic drugs for the treatment of osteoporosis, as bone mass density has been shown to increase in these patients.^18^ Paradoxically, suppression of bone resorption should lead to an increase in bone mass density. Several studies have been conducted to elucidate the effect of vitamin D on osteoclasts *in vivo*; however, the effects have been reported as both inhibitory and stimulatory, and the exact mechanisms remain unknown.^19^ In *in vitro* studies (doses of more than 1 nmol·L^−1^), 1,25(OH)_2_D_3_ has been shown to have both stimulatory as well as inhibitory effects on osteoclast activity.^19–22^

In co-culture studies of osteoblasts and hematopoietic cells, active metabolites of vitamin D has been shown to stimulate osteoclastogenesis.^23–25^ This stimulation has been shown to be an increase in RANKL production and consequently osteoclast stimulation. Hence, 1,25(OH)_2_D_3_ has been believed to directly stimulate osteoclast resorption. As the active metabolites of vitamin D has been used therapeutically, the increase in bone mass density in osteoporotic patients has been assumed to be suppression of bone resorption. Thus, the effects of 1,25(OH)_2_D_3_ on osteoclast bone resorption in *in vitro* studies seem to be opposite to *in vivo* studies. The direct effects of 1,25(OH)_2_D_3_ on osteoclast precursor and stimulation of resorption has been suggested, but the mechanism of direct action of vitamin D on bone cells and the dose of vitamin D yet to be determined.^19^

Seeking to improve the understanding of the effects of vitamin D on osteoclasts, we have examined the expression of VDR receptor in multinucleated resorbing osteoclasts in human bone tissue, as well as in osteoclast cell lysates from *in vitro* studies. We have also studied the effects of 25(OH)D_3_ and 1,25(OH)_2_D_3_ metabolites on osteoclast activity, with a particular emphasis on lower doses of 1,25(OH)_2_D_3_.

## Materials and methods

### Reagents and chemicals

All chemicals were purchased from Sigma-Aldrich (Gillingham, Dorset, UK), or as otherwise stated. 25(OH)D_3_ and 1,25(OH)_2_D_3_ (Isoscience, CertiMass, Pennsylvania, USA) were dissolved in absolute ethanol at 10^−3^ mol·L^−1^ concentration as a stock solution, and stored in light-protected glass vials at −80 °C. Working dilutions of 25(OH)D_3_ were evaluated by liquid chromatography–mass spectrometry. All sera used for tissue culture were routinely assessed for endogenous levels of 25(OH)D_3_. Recombinant human M-CSF was purchased from R&D systems Europe (Abingdon, UK) and recombinant human RANKL was purchased from PeproTech (London, UK).

### Immunohistochemistry

Human bone samples were collected from patients undergoing total hip/knee replacement under ethical approval and in compliance with the University of Oxford Musculoskeletal Biobank and Human Tissue Act ethical procedures. Cylindrical bone cores were taken longitudinally, fixed in 4% paraformaldehyde at 4 °C overnight, decalcified in 0.5 mol·L^−1^ ethylenediaminetetraacetic acid (Lonza, UK) solution over 6 weeks, and embedded in paraffin. Paraffin-embedded human bone samples (non-pathological) were kindly provided by Dr Kashima Takeshi, Pathology Department, Nuffield Orthopaedic Centre, University of Oxford. Bone samples were cut in serial sections at 5 μm and mounted on adhesive glass slides (Lecia Biosystems, Milton Keynes, UK). Slides were deparaffinised in xylene and rehydrated through a graded series of alcohols to water. Slides were washed with tris-buffered saline and Tween 20 (TBST), and endogenous peroxidase activity was quenched by 3% hydrogen peroxide for 30 min. Antigen retrieval was performed on an 85 °C hot plate using citrate buffer for 10 min. Non-specific reactivity was blocked in TBST–5% bovine serum albumin for 30 min at room temperature. Representative slides were incubated overnight at 4 °C with mouse anti-human VDR monoclonal antibody (Santa Cruz Biotechnology, Heidelberg, Germany) or for 1 h with mouse anti-human cathepsin K monoclonal antibody (Merck Millipore, Hertfordshire, UK) at a dilution of 1:200 and 1:400, respectively.

After primary antibody incubation reaction, secondary biotinylated rabbit anti-mouse antibody (Vector Laboratories, Peterborough, UK) was applied for 30 min at room temperature. Sections then were rinsed with TBST, and visualized using the avidin–biotin peroxidise diluted at 1:200 for 30 min. Sections were rinsed with TBST and treated with 3,3-diaminobenzidine (Vector Laboratories). Sections were counterstained with hematoxylin for 10 s, washed under running water for 30 s, dehydrated in ethanol, cleared in xylene, and cover slipped with Dibutylphthalate Polystyrene Xylene mounting medium. Sections were examined using an Olympus BX40 light microscope (Philadelphia, PA, USA) and photographs were captured at ×4–20 magnifications. For general morphological analysis, decalcified sections were stained with Mayer’s hematoxylin and eosin, and negative control sections were incubated with the non-immunized mouse IgG2a and IgG1 (R&D Systems, UK).

### Cell sorting

Buffy coats enriched of CD14+ monocytes were obtained from Oxford Radcliffe Biobank (Oxford, UK), and cell sorting was performed using the Magnetic-activated cell sorting (MACS) column separation kit according to the manufacturer’s instructions (Miltenyi Biotec, Surrey, UK). Buffy coats were diluted in a 1:1 ratio in α-minimal essential medium (Invitrogen, Paisley, UK), layered over Histopaque and centrifuged (750 *g*) for 25 min. The interface layer was resuspended in α-minimal essential medium and then centrifuged (600 *g*) for a further 10 min. The resultant cells were incubated with MACS CD14+ microbeads (Miltenyi Biotec) for 20 min at 4 °C, passed through MACS magnetic column separator, and resuspended in medium supplemented with 10% heat-inactivated charcoal-stripped serum (CSS; Sigma-Aldrich) and the cells were counted by hemocytometer.

### Osteoclast formation assay

To assess the extent of osteoclast formation, isolated human CD14+ cells were cultured in 24-well plates (1×10^6^) and maintained in 1 ml α-minimal essential medium-CSS with 25 ng·mL^−1^ recombinant human M-CSF (R&D Systems) and ±50 ng·mL^−1^ recombinant human sRANKL (osteoclast culture media; PeproTech). Cells were maintained in osteoclast culture media and treated every other day in the presence or absence of 25(OH)D_3_ (100 or 500 nmol·L^−1^) or 1, 25(OH)_2_D_3_ (0.1 or 0.5 nmol·L^−1^) in ethanol as vehicle (total added volume was <1% of the culture medium volume). Osteoclast culture media containing the vitamin D metabolites were changed every other day up to 6 days before RNA and 14 days for protein extraction.

After 6 days in culture, the expression of TRAP in the cultures was examined histochemically. The culture plates were rinsed in phosphate-buffered saline, fixed with 4% formaldehyde for 10 min, and rinsed in distilled water. TRAP was histochemically revealed by a simultaneous coupling reaction using Naphtol AS-BI-phosphate (Sigma-Aldrich, Dorset, UK) as a substrate and Fast violet B (Sigma-Aldrich, Dorset, UK) as the diazonium salt. The wells were then incubated for 60 min at 37 °C in the dark, rinsed three times in distilled water, and the residual activity was inhibited by 4 % NaF for 30 min. TRAP-positive cells, with more than three nuclei, were identified as osteoclasts. The number of generated osteoclasts were assessed using light microscopy. The size of the osteoclasts was determined by image analysis using the ImageJ software (National Institutes of Health, Bethesda, MD, USA).

### Osteoclast resorption assay

To assess the extent of resorption, human CD14+ cells were cultured in 96-well plates on 3 mm dentin slices. Cells were maintained in osteoclast culture media and treated every other day in the presence or absence of 25(OH)D_3_ (100 or 500 nmol·L^−1^) or 1, 25(OH)_2_D_3_ (0.1 or 0.5 nmol·L^−1^) in ethanol as vehicle. After 14 days, the dentine slices were removed from the 96-well plates, placed in 1 mol·L^−1^ NH_4_OH for 30 min, and sonicated to remove adherent cells. Dentins were rinsed in distilled water, air dried, images were captured by light microscopy, and resorption pit surface areas (μm^2^) were analyzed with image analysis software (ImageJ; http://rsbweb.nih.gov/ij/). In some experiments, cells were removed with 1 mol·L^−1^ NH_4_OH, and resorptive pits were stained with toluidine blue and analyzed by light microscopy.

### Western blotting

After 3 days of culture of osteoclast precursor and 14 days of culture of osteoclasts, cells were washed with phosphate-buffered saline and homogenized in radioimmunoprecipitation lysis buffer containing protease inhibitor. Samples were sonicated for 30 s and spun at 12 000 r·min^−1^ for 20 min at 4 °C, the supernatant was collected. and protein concentration was determined using a bicinchoninic acid protein assay kit (Pierce Biotechnology, Rockford, IL, USA). Samples (20 μg per lane) were separated using 10% acrylamide gels and blotted onto polyvinylidene difluoride membranes. Membranes were washed in TBST and blocked with TBST–5% bovine serum albumin. Samples were incubated overnight with mouse anti-human VDR monoclonal antibody (Santa Cruz Biotechnology). Subsequently, the membranes were washed in TBST and incubated with the appropriate secondary antibodies coupled with horseradish peroxidase. Immunoreactive bands were visualized and detected using an enhanced chemiluminescence kit (GE Healthcare Life Sciences, Amersham, UK). For comparison and conformation of VDR expression in human CD14+ cells, cell lysates of human (SaOS-2 and MG-63) and murine (2T3 and MC3T3) osteoblast-like cells as well as HL-60 cell lysates were loaded and examined.

### RNA extraction, complementary DNA synthesis, and RT-PCR

Human CD14+ cells were cultured in 24-well plates (1×10^6^) in osteoclast culture medium and presence or absence of 25(OH)D_3_ (100 or 500 nmol·L^−1^) or 1,25(OH)_2_D_3_ (0.1 or 0.5 nmol·L^−1^) over 6 days. Total RNA from each treatment was extracted using the RNeasy Mini kit (Qiagen, Manchester, UK) according to the manufacturer’s instructions. Nucleic acid concentrations were measured by NanoDrop ND-1000 spectrophotometer (Thermo Scientific, Loughborough, UK) at 260 nm. The absorbance ratios at 260/280 and 260/230 were used to detect any protein or organic carryover. Samples with both 260/280 and 260/230 ratios of ≥2 were used for further analysis. The integrity of total RNA purified with Qiagen RNeasy kit was also assessed using 1.5% agarose gel electrophoresis and ethidium bromide staining.

A total of 2 μg from each extract was treated with RNase-free DNase I (Thermo Scientific) for 30 min at 37 °C. Removal of genomic DNA was terminated by further heat inactivation with 100 mmol·L^−1^ ethylenediaminetetraacetic acid at 65 °C for 10 min. First strand complementary DNA synthesis of template RNA extracts was performed using a Veriti 96-well Thermal Cycler (Applied Biosystems, Warrington, UK) using Bio-Rad iScript Reverse Transcription Supermix (Bio-Rad, Hertfordshire, UK) in a final reaction volume of 40 μl according to the manufacturer’s instruction.

RT-PCR was performed using a ViiA7 system (Applied Biosytems) with commercially available lyophilized QuantiTect Qiagen primers: *vdr*, *cyp27b1*, *cyp24a1*, *nfatc1*, and *tm7sf4*. A volume of 1 μL of one-twentieth dilutions of templates were used in a total volume of 10 μl reaction in 384-well plates (Applied Biosystems) by two-step cycling (polymerase activation at 95 °C for 2 min, 40 cycles of template denaturation at 95 °C for 5 s, and primer annealing and extension at 65 °C for 30 s) using SYBR green Master Mix (SensiFAST SYBR Lo-Rox kit, Bioline, London, UK). The *C*_t_ values for treated samples were normalized to housekeeping genes *gapdh* and the relative expressions were calculated using ^ΔΔ^*C*_t_ with amplification and accuracy of 98%–100%.

### Statistical analysis

All experiments were carried out at least in triplicates and the mean±s.e.m. was calculated. Statistical analyses were carried out using SPSS version 11.0 for windows (SPSS, Chicago, IL, USA). Effects of vitamin D treatments in respect to untreated cells were compared by Student’s *t*-test and a *P* value of <0.05 was regarded to indicate a significant difference.

## Results

### Immunohistochemistry

**Figure 1:**
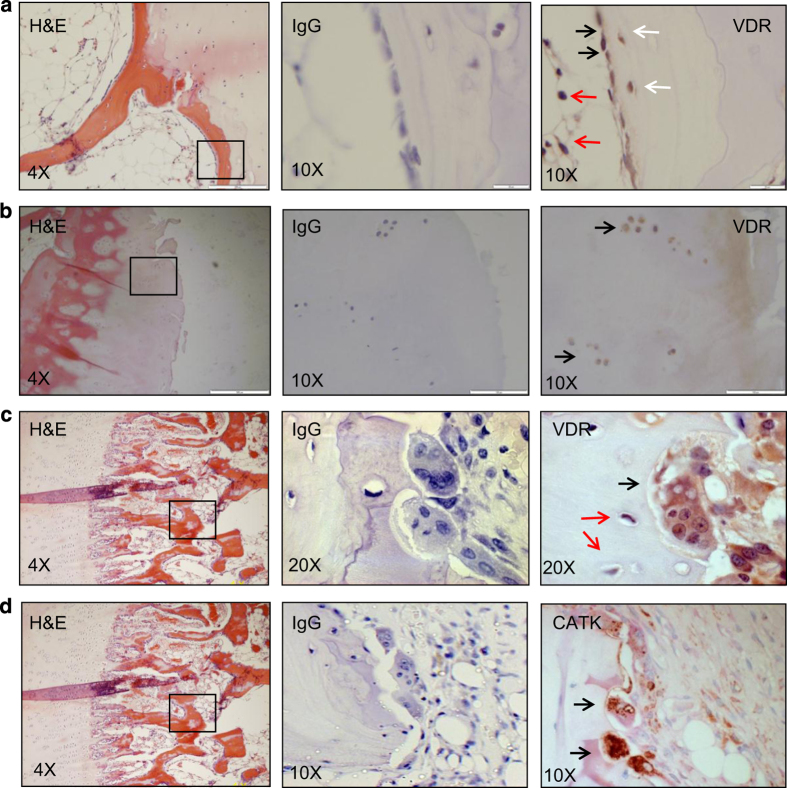
VDR is expressed by BMSCs, osteoblasts, osteocytes, chondrocytes, and osteoclasts in bone tissue. Serial sections of human bone samples, right column; hematoxylin and eosin staining, middle column; negative staining (IgG), left column; IHC of VDR (**a**–**c**), and (d) Cathepsin K (CATK). Positive cross-reactivity of VDR was observed in bone lining osteoblasts (black arrows in a), newly embedded osteocytes (white arrows in a), adipocytes (red arrows in a), chondrocytes (black arrows in b), bone resorbing multinucleated osteoclast (black arrow in c). Negative cross-reactivity was observed in mature osteocytes (red arrows in c).

VDR immunoreactivity in human bone tissue was observed in bone lining osteoblasts (Figure 1a, black arrows) and newly embedded osteocytes (Figure 1a, white arrows), but not in mature osteocytes (Figure 1c, red arrows), adipocytes (Figure 1a, red arrows), and chondrocytes (Figure 1b, black arrows). Multinucleated osteoclasts on the bone surfaces with clear lacunar pit (Figure 1c, black arrow) also showed VDR immunoreactivity. Immunohistochemical staining of serial sections revealed that osteoclasts also were immune-reactive for cathepsin K (Figure 1d, black arrows). There was no staining of VDR or cathepsin K in serial sections exposed to non-immunized mouse IgG negative (Figure 1a–d, middle images).

### Western blotting

**Figure 2:**
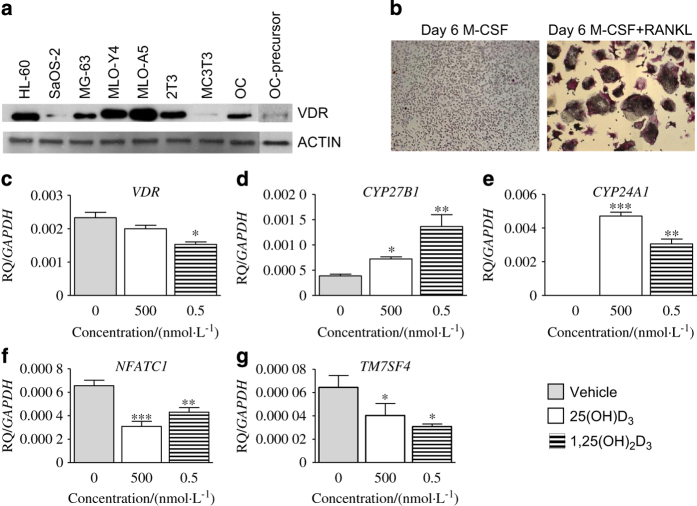
Bone cells express VDR in *in vitro* cell culture. Western blots of 20 μg extracts of human osteoblast-like cells SaOS-2, MG-63, osteocyte-like cells MLO-Y4 and MLO-A5, mouse osteoblast-like cells 2T3 and MC3T3 as well as osteoclasts (OC) cell lysates (**a**). Human CD14+ cells were cultured in 24-well plates (1×10^6^) in osteoclast culture medium (RANKL–M-CSF) and presence or absence of 25(OH)D_3_ (500 nmol·L^−1^) or 1,25(OH)_2_D_3_ (0.5 nmol·L^−1^) over 6 days. (**b**) Representative image of TRAP staining of CD14+ cells in response to M-CSF or RANKL–M-CSF on day 6. Effects of vitamin D metabolites on the relative quantity (RQ) of vitamin D-related signature gene expression; *VDR* (**c**), *CYP27B1* (**d**), *CYP24A1* (E), *NFATC1* (**f**), and *TM7SF4* (**g**). The magnitude of increase in expression of each gene is normalized to the housekeeping gene *GAPDH*. Error bars: mean±s.e.m. of triplicates (Student’s *t*-test), *P<0.05, **P<0.01, and ***P<0.001 for comparisons between vitamin D metabolite treatments against control (gray). Note that Western blot of osteoclast (OC) precursor in **a** was run separately.

To assess the expression of VDR *in vitro* cell cultures, cell lysates of human osteoclasts as well as other in-house bone cell lysates were assessed by western blotting. VDR protein was expressed in all cell lysates; human osteoclast precursor, differentiated osteoclasts, human and mouse osteoblast, and mouse osteocyte-like cell lysates as well as HL-60 control sample (Figure 2a), with highest expression in MLO-Y4 and MLO-A5 osteocyte-like cell lines and lowest expression in SaOS-2, MC3T3 osteoblasts, and human osteoclast precursor.

### Effect of vitamin D metabolites on vitamin D-related genes

The effects of vitamin D metabolites during osteoclastogenesis on vitamin D metabolic pathway genes was examined by qRT-PCR. Initially, we tested the effects of M-CSF or RANKL–M-CSF for the generation of osteoclasts, where 6 days of RANKL–M-CSF generated maximal number of multinucleated osteoclasts (Figure 2b). The data revealed that osteoclasts express *VDR*, *CYP27B1*, and *CYP24A1* at messenger RNA level. *VDR* messenger RNA expression was not altered in response to 500 nmol·L^−1^ 25(OH)D_3_ (Figure 2c), whereas treatments of osteoclast with 0.5 nmol·L^−1^ 1,25(OH)_2_D_3_ suppressed *VDR* expression (Figure 2c; *P*<0.05). Treatment of human CD14+ cells with either 500 nmol·L^−1^ 25(OH)D_3_ or 0.5 nmol·L^−1^ 1,25(OH)_2_D_3_ significantly upregulated *CYP27B1* (Figure 2d; *P*<0.05 and *P*<0.01, respectively) and *CYP24A1* (Figure 2e; *P*<0.001 and *P*<0.01, respectively). Both 500 nmol·L^−1^ 25(OH)D_3_ or 0.5 nmol·L^−1^ 1,25(OH)_2_D_3_ significantly suppressed *NFATC1* (Figure 2f; *P*<0.001 and *P*<0.01, respectively) and *TM7SF4* (Figure 2g; *P*<0.05) expression in osteoclasts.

### Osteoclast function in response to vitamin D metabolites

**Figure 3:**
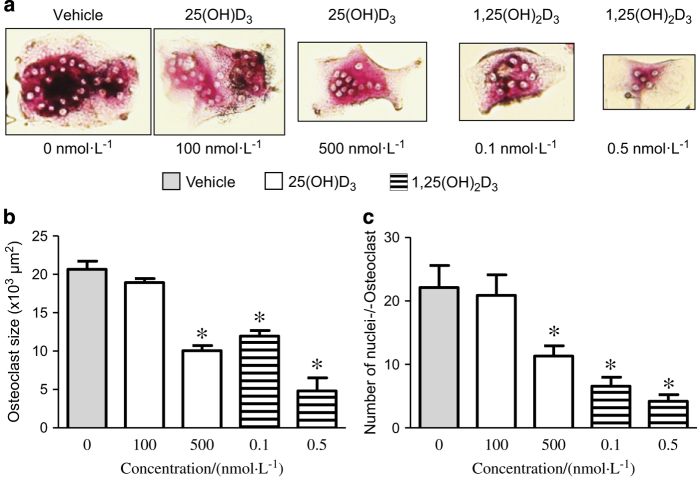
Osteoclast size and number of nuclei per osteoclast are reduced by vitamin D metabolites. Human CD14+ cells were cultured in 96-well plates (0.25×10^6^) in osteoclast culture medium (RANKL–M-CSF) and presence or absence of 25(OH)D_3_ (100 and 500 nmol·L^−1^) or 1,25(OH)_2_D_3_ (0.1 and 0.5 nmol·L^−1^) over 6 days. After 6 days in culture, the expression of TRAP in the cultures was examined histochemically (**a**). The size of each osteoclast (**b**) (μm^2^) and number nuclei per cell (**c**) were assessed and determined by image analysis using the ImageJ software (National Institutes of Health). Error bars: mean±s.e.m. of triplicates (Student’s *t*-test), *P<0.001 for comparisons between vitamin D metabolite treatments against control (gray).

Osteoclast size was not affected in response to concentration of 100 nmol·L^−1^ of 25(OH)D_3_ (Figures 3a and b), whereas high concentration of 25(OH)D_3_ (500 nmol·L^−1^) significantly reduced osteoclast size (Figures 3a and b: *P*<0.001). Both doses of 0.1 and 0.5 nmol·L^−1^ of 1,25(OH)_2_D_3_ significantly reduced osteoclast size (Figure 3a and b: 0.1 and 0.5 nmol·L^−1^; *P*<0.001).

The number of nuclei per osteoclast that is an indication of how many osteoclasts precursors may have fused together was examined in response to vitamin D metabolites. The number of nuclei per osteoclast was not changed in response to 100 nmol·L^−1^ 25(OH)D_3_, but significantly decreased in response to 500 nmol·L^−1^ of 25(OH)D_3_ (Figure 3c). Concentrations of 0.1 and 0.5 nmol·L^−1^ 1,25(OH)_2_D_3_ dose-dependently decreased the number of nuclei per osteoclast (Figure 3c).

**Figure 4:**
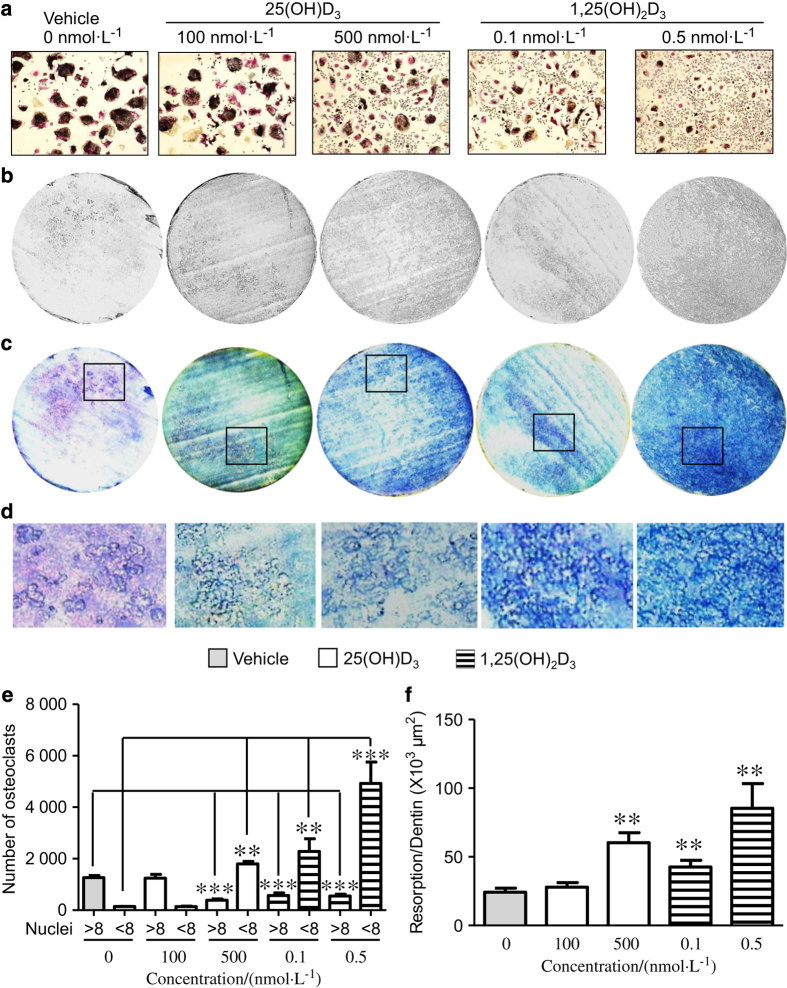
**Vitamin D metabolites increase osteoclast number and resorption.** Human CD14+ cells were cultured in 24-well plates (1×10^6^) in osteoclast culture medium (RANKL–M-CSF) or 96-well plates (0.25×10^6^) on 3 mm dentin slices in the presence or absence of 25(OH)D_3_ (100 and 500 nmol·L^−1^) or 1,25(OH)_2_D_3_ (0.1 and 0.5 nmol·L^−1^) over 6 days for TRAP staining or 14 days for resorption. After 6 days, the expression of TRAP in the cultures was examined histochemically, images were taken by Zeiss AX10 microscope (Carl Zeiss, Jena, Germany) and number of positive cells (**a**) with greater or less than eight nuclei were counted manually (**e**). After 14 days, the dentine slices were removed from the 96-well plates, placed in 1 mol·L^−1^ NH_4_OH for 30 min and sonicated to remove adherent cells. Dentins were rinsed in distilled water, air dried, and images were captured by light microscopy (**b**). Dentins were stained with toluidine blue, photographed (**c**), and resorption areas per dentin slice (**f**) (μm^2^) were analyzed with ImageJ software (National Institutes of Health). (**d**) High magnifications (×10) of dentins in **c**. Error bars: mean±s.e.m. of triplicates experiments (Student’s *t*-test), **P<0.01, and ***P<0.001 for comparisons between vitamin D metabolite treatments against control (gray).

TRAP staining of vitamin D-treated osteoclasts (Figure 4a) revealed that only 500 nmol·L^−1^ of 25(OH)D_3_ increased osteoclast cell number (Figure 4e; *P*<0.05), whereas active metabolites of 1,25(OH)_2_D_3_ significantly increased osteoclast cell number (with ≤8 nuclei) dose-dependently (Figure 4e; 0.1 nmol·L^−1^: *P*<0.05, 0.5 nmol·L^−1^: *P*<0.001, respectively). A concentration of 500 nmol·L^−1^ of 25(OH)D_3_ or 0.1 nmol·L^−1^ of 1,25(OH)_2_D_3_ significantly reduces osteoclasts with ≥8 nuclei (Figure 4e; *P*<0.001). To determine the functional activity of osteoclasts in response to vitamin D metabolites, their resorptive activities on dentine discs were examined (Figures 4b-d). Resorption was not altered when cells were treated with 100 nmol·L^−1^ 25(OH)D_3_; however, resorption was significantly increased in response to 500 nmol·L^−1^ 25(OH)D_3_ (Figure 4f). When cells were treated with 1,25(OH)_2_D_3_ both concentrations of 0.1 and 0.5 nmol·L^−1^ significantly increased resorption (Figure 4f). Supra-physiological concentrations of 25(OH)D_3_ (≥1 000 nmol·L^−1^) or 1, 25(OH)_2_D_3_ (≥1 nmol·L^−1^) metabolites abolished osteoclast activities (data not shown).

## Discussion

This study found that osteoclasts in bone tissue as well as *in vitro* cell cultures express VDR, and aimed to establish their responsiveness to various concentrations of vitamin D. As shown by qRT-PCR, osteoclastogenesis was stimulated in response to 500 nmol·L^−1^ of 25(OH)D_3_ and 0.1–0.5 nmol·L^−1^ of 1,25(OH)_2_D_3_, upregulating *CYP27B1* and *CYP24A1*, but osteoclast fusion markers NFATC1 and TM7SF4 were suppressed by vitamin D metabolites osteoclast number and resorption activity were also increased in response to 25(OH)D_3_ and 1,25(OH)_2_D_3_. Both 25(OH)D_3_ and 1,25(OH)_2_D_3_ reduced osteoclast size and number when co-treated with RANKL and M-CSF. Lower concentrations (100 nmol·L^−1^) of 25(OH)D_3_ had no effect on osteoclast activity.

There is growing evidence that bone is an autocrine organ for vitamin D metabolism.^21,26^ The direct effect of circulatory 25(OH)D_3_ on bone mineralization has been shown both in human^27^ and rodent^28^ studies as well as the effect of 25(OH)D_3_ and 1,25(OH)_2_D_3_ on human and rodent osteoblast cells in *in vitro* studies.^2^ Detection of VDR in multinucleated resorbing osteoclasts with distinct resorption lacunae in the current study suggests that in addition to regulating osteoblast mineralization, other cells such as osteoclasts are also direct target of vitamin D metabolites.

Current evidence suggests that osteoblasts, through their VDR expression, mediate the action of 1,25(OH)_2_D_3_ on osteoclast activities via RANKL and osteoprotegerin.^29^ In the current study, we observed that the level of CYP27B1 transcript expression was increased during osteoclast differentiation when treated with 25(OH)D_3_, M-CSF, and RANKL. This observation suggests that osteoclast cells as being an extra-renal target for 1,25(OH)_2_D_3_ synthesis and action and this is consistent with reduced osteoclast TRAP positivity and osteoclast number in CYP27B1 null mouse.^30^ CYP24A1 is an enzyme that catalyzes the initial steps in inactivation of 1,25(OH)_2_D_3_. An increase in the level of this enzyme during osteoclastogenesis initially confirms the action of vitamin D metabolites in osteoclasts and furthermore determine both the level and duration of osteoclast response to 1,25(OH)_2_D_3_. These observations are consistent with low level of VDR protein expression in osteoclast precursor compared with higher VDR protein expression in mature osteoclasts as shown by western blotting.

Osteoclast fusion has been shown previously to be regulated by NFATC1 (ref. 31) and TM7SF4,^32^ and a decrease in the expression of these markers in response to vitamin D metabolites in our study suggests that osteoclast fusion is inhibited. These data are consistent with a decrease in osteoclast size and number of nuclei per each osteoclast. Our data indicate that an increase in osteoclast resorption is due to less fusion resulting in more small osteoclasts in treated conditions rather than fewer larger multinucleated osteoclasts in control samples.

The present study provides immunohistochemical evidence in human bone tissue that osteoclasts express VDR, possibly for metabolism of 25(OH)D_3_ as well as being responsive to 1,25(OH)_2_D_3_ active metabolite. Consistent with our finding, other studies have shown increased expression of VDR messenger RNA in osteoclast resorption lacunae from samples of hyperparathyroidism, osteoclastoma, or pagetic bone.^33^ It was of interest that beside osteoclasts, other bone cells, such as osteoblasts, newly embedded osteocytes, and adipocytes, as well as chondrocytes, also express VDR, confirming that bone cells could be a target for direct effect of vitamin D metabolites. The tissue expression of VDR by osteoclasts was consistent with its expression in pre-osteoclasts and osteoclasts derived from CD14+ cell lysates from *in vitro* cell cultures.

Osteoclast cells have been shown to express *CYP27B1*, metabolizing significant levels of 1,25(OH)_2_D_3_ (0.5 nmol·L^−1^), stimulating osteoclastogenesis, when treated with high concentrations (500 nmol·L^−1^) of 25(OH)D_3_.^7^ An increase in osteoclast cell number and resorption activity in response to 500 nmol·L^−1^ doses of 25(OH)D_3_ in our data is probably due to an increase in 1,25(OH)_2_D_3_ metabolism that is shown elsewhere.^7^ The dose-dependent increase in bone resorption observed with 0.1 and 0.5 nmol·L^−1^ concentrations of 1,25(OH)_2_D_3_ active metabolite, however, is in stark contrast to previous findings of a decline in bone resorption by 1,25(OH)_2_D_3_.^19–21^ Our findings also conflict with previous reports on 25(OH)D_3_. Osteoclast resorption has been reported in response to 25(OH)D_3_ or its active metabolites, whereas we have shown an increase in osteoclast in response to both metabolites.^7,19^

This opposite effect could be due to a number of points of difference in the materials used for this study. Of particular, importance is the substantially lower doses of 1,25(OH)_2_D_3_ with respect to the literature, which reports doses in the range of 1–100 nmol·L^−1^.

Related to dosage was the use of CSS and vitamin D metabolites that were pre-screened by liquid chromatography–mass spectrometry for metabolite concentration and the effects of sera+M-CSF alone. After observing surprising TRAP staining results in the control samples in a number of CSS with M-CSF treatment alone, we examined all in-house sera for 25(OH)D_3_ content by liquid chromatography–mass spectrometry, finding levels of 25(OH)D_3_ up to 150 nmol·L^−1^ (fetal calf serum). The CSS used in this study was the only tested sera with no detectable metabolite levels and no TRAP staining was observed in the presence of M-CSF alone. Extending the mass spectroscopy measurements to metabolite supplies, we further observed two batches of purchased 25(OH)D_3_ and 1,25(OH)_2_D_3_ in which vitamin D metabolites could not be detected.

Another source of difference to the majority of previous studies may be the use of CD14+ cells rather than PBMCs, and combined rather than separate treatments of RANKL and M-CSF. This selective isolation of osteoclast precursor cells that are committed to differentiate in response to RANKL–M-CSF factors, may produce a different ‘bulk’ response to PBMCs (containing ~10% precursor).

In conclusion, resorbing osteoclast cells in human bone tissue express VDR, suggesting that vitamin D metabolites could directly affect osteoclast function. Treatments of osteoclasts derived from CD14+ cells with vitamin D metabolites increased osteoclast cell number and resorption. Concentrations of 1,25(OH)_2_D_3_ in the 0.1–0.5 nmol·L^−1^ range dose-dependently increase osteoclast cell number and resorption.
